# B waves: a systematic review of terminology, characteristics, and analysis methods

**DOI:** 10.1186/s12987-019-0153-6

**Published:** 2019-10-15

**Authors:** Isabel Martinez-Tejada, Alexander Arum, Jens E. Wilhjelm, Marianne Juhler, Morten Andresen

**Affiliations:** 1 Clinic of Neurosurgery, Copenhagen University Hospital, Rigshospitalet, Copenhagen, Denmark; 2 Department of Health Technology, Technical University of Denmark, Kongens Lyngby, Denmark

**Keywords:** Intracranial pressure, B waves, Slow waves, Vasogenic waves

## Abstract

****Background**:**

Although B waves were introduced as a concept in the analysis of intracranial pressure (ICP) recordings nearly 60 years ago, there is still a lack consensus on precise definitions, terminology, amplitude, frequency or origin. Several competing terms exist, addressing either their probable physiological origin or their physical characteristics. To better understand B wave characteristics and ease their detection, a literature review was carried out.

****Methods**:**

A systematic review protocol including search strategy and eligibility criteria was prepared in advance. A literature search was carried out using PubMed/MEDLINE, with the following search terms: *B waves + review filter*, *slow waves + review filter*, *ICP B waves*, *slow ICP waves*, *slow vasogenic waves*, *Lundberg B waves*, *MOCAIP*.

****Results**:**

In total, 19 different terms were found, *B waves* being the most common. These terminologies appear to be interchangeable and seem to be used indiscriminately, with some papers using more than five different terms. Definitions and etiologies are still unclear, which makes systematic and standardized detection difficult.

****Conclusions**:**

Two future lines of action are available for automating macro-pattern identification in ICP signals: achieving strict agreement on morphological characteristics of “traditional” B waveforms, or starting a new with a fresh computerized approach for recognition of new clinically relevant patterns.

## Background

Intracranial pressure (ICP) monitoring plays an important role in the management of patients with many neurological and neurosurgical disorders. In the 1960s, Lundberg described typical macro-patterns: A, B and C waves [1]. B waves were defined as short repeating elevations in ICP (10–20 mmHg) with a frequency of 0.5–2 waves/min. These classic B wave patterns may be seen in ICP monitoring in intensive care unit settings (ICU), but ICP is also monitored in a large number of brain diseases covering a spectrum from acute and subacute ICU settings to elective outpatient follow-up. Today a large proportion of patients undergo ICP monitoring for milder degrees of disease where pathological patterns are not as prominent. In such scenarios, wave patterns are still called B waves but differ in amplitude and visual appearance from those defined by Lundberg. Such ‘uncharacteristic’ B waves are often smaller in amplitude and appear as an irregular pattern, but they have not yet been formally classified. The current paper uses B waves as an encompassing umbrella for all variations.

The source of B waves is unknown and although they are mostly associated with cerebral dysfunction, their clinical significance is unclear, as they may also appear as normal physiological phenomena [2, 3]. Their source is most commonly related to vasogenic activity, but an origin from a neuro-pacemaker system has also been suggested [4]. This diverging information poses a challenge to a consensus for a general description of B waves and their quantification, hindering their identification during diagnosis and treatment of different diseases categories. Because of these difficulties, clinical practice outside specialized centers with a focus on ICP-related research is currently largely restricted to readings of mean ICP.

Identification of waveform abnormalities by simple visual inspection is still a common clinical practice. This has an obvious bias from reliance on personal empiric experience and raises questions of interobserver reproducibility. Automated and standardized detection of B wave patterns would increase the usefulness in both clinical and research settings. This automated detection is only possible if the waveform morphological characteristics are clearly defined; preferably by consensus in the scientific community. A systematic quantitative detection system could allow for identification of B wave variations and other ‘non-Lundberg’ patterns, replacing traditional visual inspection.

The aim of this study was to assess the various terms and definitions used to describe classical B waves in order to highlight the lack of consensus in terms of terminology and morphological characteristics, frequency and amplitude. Therefore, a systematic review was carried out to summarize the different terminologies and definitions regarding B waves and the methods used for B wave identification.

## Methods

**Fig. 1:**
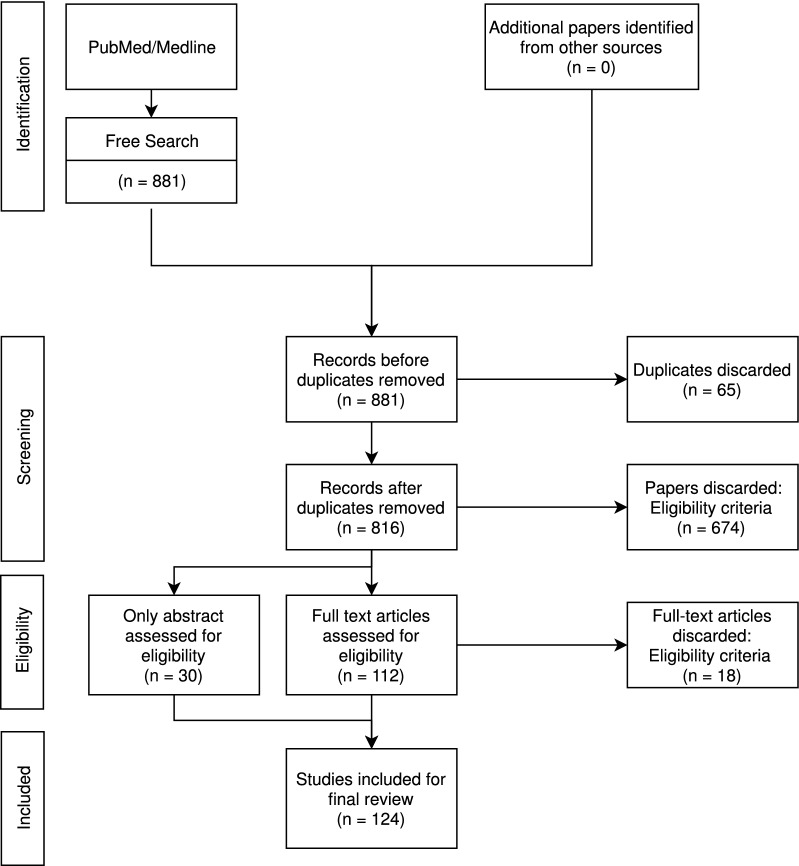
Modified PRISMA 2009 flow diagram. Systematic literature search and selection process overview. Given that the goal of this literature review was to give an insight into the different terminologies and definitions of B waves, only articles specifically mentioning B waves or related terms were included in the study selection. As an example: *slow waves of ABP* was not included. Papers simultaneously published by different journals were considered as duplicates and also excluded. The remaining articles (n = 124) were thoroughly examined and included in this study following the PRISMA flow-diagram. Terminologies, definitions, and methods were identified individually by two independent reviewers and categorized according to a predefined protocol. Disagreements were resolved by consensus. No importance was given to the order of words, *ICP B waves* was treated equally to *B ICP waves*. Hyphens were removed, *B-waves* were grouped together with *B waves*. *Of* and *in* were disregarded, *slow waves of ICP* were registered as *slow waves ICP*. Only terminologies associated with ICP B waves were included (i.e. *slow waves of ABP* was not included). Terminologies in singular form were registered as plural, *B wave* was registered as *B waves*. Terms used less than three times were categorized as *other*

Relevant studies were identified by a single reviewer using the online database PubMed/Medline. The diagram in Fig. 1 gives an overview of the literature search based on the PRISMA systematic review methodology [5]. Studies were selected if they included the key terms *slow vasogenic waves*, *Lundberg B waves*, *slow ICP waves*, *ICP B waves*, *MOCAIP*, *B waves + review filter*, and *slow waves + review filter*. A total sum of 816 paper abstracts were screened initially for content relevance and 124 papers were included in the search review.

## Results

### Terminologies

**Fig. 2:**
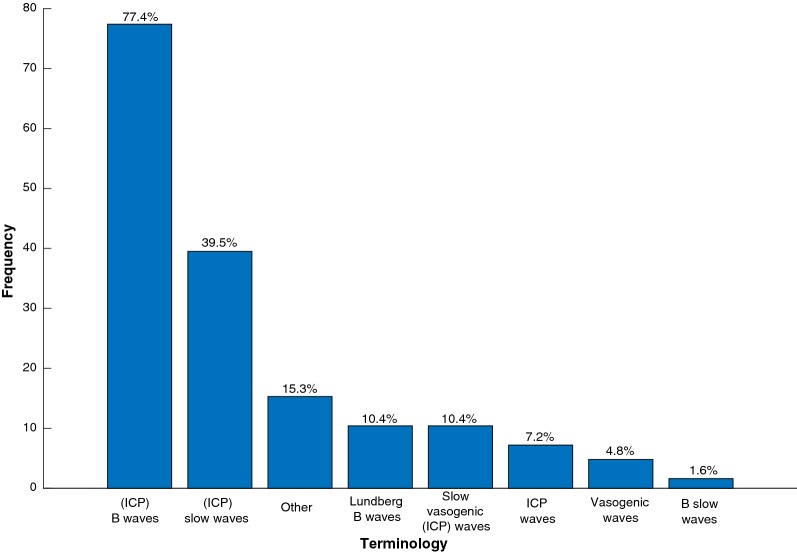
Frequency of terminology usage in the reviewed papers. The term *B waves* was used in most articles, followed by *slow waves* and *ICP slow waves*

A total of 19 terminologies were found to describe B waves in the reviewed papers (Table 1). The most common terms being *B waves* and *(ICP) slow waves* (Fig. 2). Nine articles used four or more terms to refer to B waves. The choice of terminology is often related to the ongoing etiology discussion: 22 articles include the word *vasogenic* thereby implying cerebrovascular changes as the origin of the waves. Raftopoulos [6], Santamarta [7], Yokota [8], and Kasprowicz [9], defined further subgroups in order to clarify the sources underlying the presence of B waves (Table 2).

**Table 1:** Summary of reviewed B waves terminology and characteristics

Article	Terminology								Frequency (waves/min)	Analysis tool	Additional comments
	(ICP) slow waves	ICP waves	(ICP) B waves	Lundberg B waves	B slow waves	Vasogenic waves	Slow vasogenic (ICP) waves	Other			
Spiegelberg et al. [26]			X				X		0.5–2 0.33–3	Cross-correlation	
Lalou et al. [12]	X		X			X			0.3–4	Spectral analysis	
Czosnyka et al. [27]	X						X				
Cabella et al. [28]	X										
Kojoukhova et al. [29]			X							Visual inspection	
Lalou et al. [30]	X		X			X	X	X	0.3–4	Spectral analysis	Slow wave
Czosnyka et al. [31]	X						X		0.33–3		
Santamarta et al. [7]	X		X						0.5–2		4 types, N:5
Hamilton et al. [32]	X		X	X							
Moyse et al. [33]	X										
Lu et al. [34]	X								0.5–3	Multiscale entropy	
Antes et al. [35]	X		X	X				X	0.5–3		Lundberg wave
Horcajadas Almansa et al. [36]			X								
Horcajadas Almansa et al. [37]			X						0.5–2	Visual inspection	
Varsos et al. [38]	X								0.33–3		
Cordero Tous et al. [39]			X								
Weerakkody et al. [40]	X						X	X	0.3–2	Visual inspection	Vasogenic ICP wave
Lewis et al. [41]	X		X								
Budohoski et al. [42]	X			X			X		0.3–3		
Elixmann et al. [43]			X						0.5–3		N:3
Smielewski et al. [44]	X		X				X				
Jetzki et al. [45]			X						0.5–2		
Kasprowicz et al. [9]	X		X						0.5–2	MOCAIP	3 types
Hamilton et al. [15]	X									MOCAIP	
Hu et al. [16]			X							MOCAIP	
Kim et al. [46]							X				
Shahsavari et al. [47]	X										
Horcajadas Almansa et al. [48]			X						0.5–2	Visual inspection	
Asgari et al. [49]			X								
Weerakkody et al. [50]	X		X	X		X	X		0.5–3	Spectral analysis	
Weerakkody et al. [51]	X	X	X						0.5–2 0.5–3		
Eide et al. [52]		X	X	X							
Hu et al. [17]	X										
Kasprowicz et al. [53]	X		X						0.5–2	MOCAIP	
Ursino et al. [54]			X								
Hamilton et al. [55]	X		X							Visual inspection	N:20, O:30
Lee et al. [56]			X						0.2–3		
Damasceno et al. [57]			X								
Czosnyka et al. [58]	X								0.3–3	Wavelet analysis	
Brady et al. [59]	X		X								
Stevens et al. [60]			X					X			Hypertensive ICP spikes
Petrella et al. [61]	X		X					X	0.33–3	Spectral analysis	Lundberg ICP B wave
Schuhmann et al. [62]	X		X			X		X		Spectral analysis	Vasogenic intracrnial wave
Minns et al. [63]	X		X					X		Visual inspection	Slow ICP pressure wave
Jantzen et al. [64]			X	X							
Geocadin et al. [65]			X						0.5–2		N:3
Czosnyka et al. [66]	X						X		0.33–3	Spectral analysis	
Wang et al. [67]	X										
Delavallee et al. [68]			X								
Czosnyka et al. [69]	X								0.33–3		
Guendling et al. [70]	X								0.33–3		
Czosnyka et al. [71]	X		X								
Schmidt et al. [72]			X							Visual inspection	
Agren-Wilsson et al. [73]			X								
Lescot et al. [4]			X	X				X	0.5–2		Lundberg ICP B wave
Balestreri et al. [74]	X		X				X	X	0.33–3		Vasogenic ICP wave
Stephensen et al. [75]			X						0.5–2	Spectral analysis and amplitude	
Czosnyka et al. [76]	X								0.2–3		
Fountas et al. [77]	X				X			X	0.5–2		ICP slow B wave
Czosnyka et al. [78]	X		X			X	X		0.33–2	Spectral analysis and amplitude	
Momjian et al. [14]	X		X	X			X	X	0.33–3	Spectral analysis	Lundberg ICP B wave
Lenfeldt et al. [79]			X						0.5–2		N:1
Edsbagge et al. [80]			X							Spectral analysis	
Balestreri et al. [81]	X		X						0.33–3	Spectral analysis	
Ragauskas et al. [82]	X				X			X	0.3–2		
Strik et al. [83]	X		X						0.5–3	Spectral analysis	
Daley et al. [84]		X	X						0.5–2	Spectral analysis	
Stephensen et al. [85]		X	X								
Poca et al. [86]			X								
Lemaire et al. [87]	X	X	X	X					0.5–2		
Czosnyka et al. [88]	X	X	X			X			0.5–3		
Walter et al. [89]			X						0.5–2	Spectral analysis	
Schmidt et al. [90]	X								0.5–2		
Eklund et al. [23]			X						0.5–2	Spectral analysis and amplitude	
Vanneste et al. [91]			X						0.5–2		
Schoeman et al. [92]			X						0.5–2	Visual inspection	
Schuhmann et al. [93]			X								
Czosnyka et al. [94]			X							Spectral analysis	
Droste et al. [95]			X						0.5–2	Visual inspection	
Qureshi et al. [96]			X						1–2		N:3
Czosnyka et al. [97]	X							X			Slow spontaneous waves in ICP
Czosnyka et al. [98]	X	X									
Newell et al. [99]			X						0.5–2		
Lemaire et al. [100]	X		X						0.5–2		
Steinmeier et al. [101]		X	X						0.5–2		
Hanlo et al. [13]			X						3–4		O:15
Krauss et al. [102]			X						0.5–2	Visual inspection	
Wayenberg et al. [103]			X						0.5–3		
Lemaire et al. [104]	X		X						0.5–3	Spectral analysis	
Krauss et al. [105]			X						0.5–2		
Droste et al. [106]			X							Visual inspection	
Takeda et al. [107]			X								
Newell et al. [108]		X	X						0.5–2	Spectral analysis	
Raftopoulos et al. [6]			X						0.5–3		
Sahuqillo et al. [109]			X								N:10
Hara et al. [110]			X						0.5–2	Spectral analysis	
Handa et al. [111]]			X						0.5–2		
Yokota et al. [8]			X						0.5–2	Visual inspection	
Takeuchi et al. [112]			X								
Yokota et al. [113]			X							Visual inspection	
Sato et al. [114]								X			Lundberg’s B
Maeda et al. [115]			X								
Gjerris et al. [116]			X								
Hayashi et al. [117]			X						0.5–2		
Schoeman et al. [118]			X							Visual inspection	
Kosteljanetz et al. [119]			X								
Hayashi et al. [120]			X						0.5–2		
Brock et al. [121]			X								
Kuchiwaki et al. [122]								X			B type pressure wave
Tamaki et al. [123]			X								
Auer et al. [124]			X						0.5–2		
Terzano et al. [125]				X				X			Lundberg B-type CSF pressure waves
Gjerris et al. [126]			X						1–2		N:5
Tomei et al. [127]			X								
Kaye et al. [128]			X								N:10
Bilz et al. [129]			X								
Munari et al. [130]	X							X			Pressure waves of the Lundberg type b
Guieu et al. [131]			X	X				X	0.5–2		type B pressure waves
Wilkinson et al. [132]			X								
Liguoi et al. [133]			X								
Fuentes et al. [134]			X								
Munari et al. [135]			X	X				X			Lundberg’s B waves
Hayashi et al. [136]			X								N:15, O:45
Martin et al. [2]				X							
Total sum	49	9	96	13	2	6	13	19			

Table summarizing the main terms and characteristics used to describe B waves with articles sorted based on year of publication. Under *terminology*, terms that appeared more than five times are given their own column. Less frequent terms are included under *other* and the term is listed under *additional comments*. The column *frequency* describes the occurrence in waves/min of the B waves as described in the corresponding article, while the column *analysis tool* lists the methodology used to detect B waves. Under *additional comments*, besides including other terms used to refer to the B waves, extra notes are added: N with an associated number value: Lower amplitude value of X mmHg. O with an associated number value: Upper amplitude value of X mmHg. N types: B waves subclassification into N subgroups

**Table 2:** Major morphological B wave subclasses

	Term	Shape	Plateau	Frequency (waves/min)	Amplitude (mmHg)
Raftopolous et al. [6] Santamarta et al. [7]	Small symmetrical wave (SSW)	Symmetrical	No	0.36–5	< 10
	Great symmetrical wave (GSW)	Symmetrical	No	0.36–5	> 10
	Intermediate wave (IW)	Asymmetrical	No	0.33–1.67	6–34
Kasprowicz et al. [9]	Slow symmetrical ICP wave	Symmetrical	No	–	–
	Slow asymmetrical ICP wave	Asymmetrical	No	–	–
	Slow ICP B with plateau phase	Symmetrical	Yes	–	–
Yokota et al. [8]	Type II episodic B-wave	–	–	–	25–75
	Type III persistent, high pressure B-wave	–	–	0.5–2	40–100
	Type IV continuous, regular B-wave	–	–	0.5–2	10–30

### Characteristics

B waves were identified based on two major wave parameters: frequency and amplitude. Frequency is the number of waves that fit into a certain time period, usually measured as waves per minute and 27% of the papers defined a frequency of 0.5–2 waves/min, as originally defined by Lundberg [1]. To accommodate B waves of a lower frequency, the term *slow* was introduced [10]. The term *slow waves* was then used to define waves with a frequency window of 0.33 to 3 waves/min [11]. Two other papers extended the frequency upper limit to 4 waves/min [12, 13].

As mentioned, B waves can also be characterized by their amplitude. Lundberg defined a maximum amplitude of 50 mmHg back in the 1960s. Under pathological conditions, this level of elevation is less often seen to such an extent today, and B waves with lower amplitudes are more likely to be present. As an example, lower amplitude B waves are present in cases of normal pressure hydrocephalus, where the occurrence of B waves is not related to high ICP [14].

**Fig. 3:**
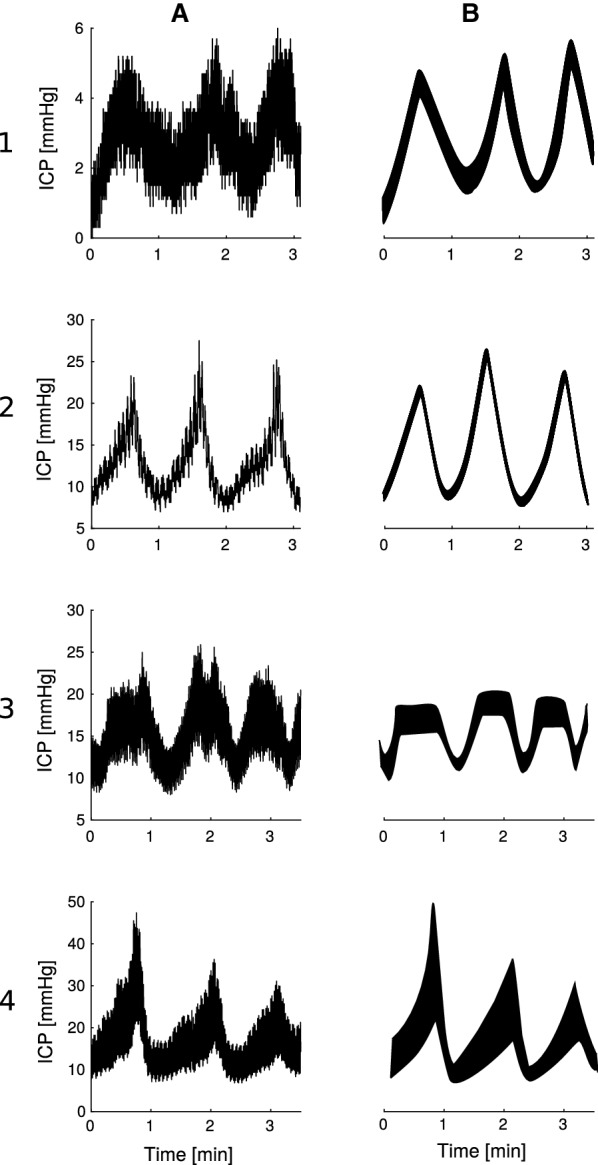
Presentation of different B waves sub-classification patterns. Each is illustrated by two computer-generated examples: column A simulating ICP recordings and column B showing an artistic rendering. Examples on rows 1 and 2 exhibit B waves with symmetrical shape and amplitude lower and higher than 10 mmHg, respectively. Examples of row 3 correspond to symmetrical B waves with plateau. The last row shows examples for asymmetrical B waves. The time-scale used in all examples is minutes

### Sub-classification

In addition to frequency and amplitude, two other parameters are generally defined for the analysis of B waves. B waves can also be characterized by their shape and whether a plateau phase is present or not. The shape is considered symmetrical if the duration of ascending and descending phases is the same. If the ascending phase is longer, then the shape is asymmetrical. The use of these parameters gives rise to different subclasses within B waves (Table 2). All subclasses fit into the traditional definition of B waves with an extended frequency spectrum, but mainly differ in their morphological characteristics (Fig. 3).

Besides these four parameters, Raftopolous et al. and Santamarta et al. also use the duration of the ICP wave to characterize B waves. They distinguish between three morphological subclasses: (1) small symmetrical waves with an amplitude below 10 mmHg, (2) great symmetrical waves with an amplitude above 10 mmHg, and (3) intermediate waves, with the same frequency as symmetrical waves but an amplitude similar to plateau waves [6, 7].

Kasprowicz et al. describe three subcategories of B waves based on the investigation of their unique shape: (1) symmetrical ICP B waves, (2) asymmetrical ICP B waves, and (3) slow ICP B waves with plateau phase. They show how the different subtypes of B waves are related to changes in the ICP pulse shape, which indicate that each has a unique origin [9]. Similarly, Yokota et al. also suggest the existence of three subgroups but from the analysis of ICP amplitude and occurrence: (1) episodic B-waves, (2) persistent, high pressure B-waves, and (3) continuous, regular B-waves, and that these patterns may better distinguish between different origins of ICP waves [8].

The intermediate waves described by Raftopolous et al. [6] and Santamarta et al. [7] contain amplitudes similar to plateau waves, Kasprowicz et al. [9] describe B waves with a plateau phase, and Yokota et al. [8] describe persistent high pressure B waves. It is noteworthy that all sub-classification attempts contain a B wave subtype with plateau-like features. This raises the question whether there is a continuous transition from B waves to plateau waves or whether they have different etiology.

To summarize, B waves are categorized into different subclasses if they have distinct shapes *and/or* if their amplitude is different. These sub-classification attempts may be used as supplementary evidence that the classical waveform categories do not adequately address waveforms identified in clinical practice today.

### Analysis tools

32% of the papers had an explicitly stated analysis method. While traditionally the most common analytical method used was either spectral analysis (40%) or spectral analysis with an amplitude threshold (7%), there is now an increasing tendency (10%) to detect B waves using trained machine learning algorithms, as observed in more recently published papers [9, 15, 16]. These algorithms use as input morphological features extracted from the ICP pulse wave via the Morphological clustering and analysis of ICP pulse (MOCAIP) algorithm. Thus, instead of defining B waves in terms of amplitude and frequency, they define them according to different morphological parameters of the pulse wave. These parameters are based on the three subpeaks ($P_1$, $P_2$, and $P_3$) of the pulse wave: systolic peak, tidal peak, and dicrotic peak, respectively [9]. Examples of these ICP pulse metrics include the amplitude of the subpeaks, the latency between subpeaks, and the start of the ICP pulse wave and the pulse wave period, among others [17].

## Discussion

ICP arises from pressure contributions from the brain, the heart, and the cerebrospinal fluid (CSF) inside the skull [18]. ICP is monitored invasively with a pressure transducer inserted either intracranially (subdural, epidural, intraparenchymal or intraventricular placements) or in the spinal compartment (lumbar puncture). As the brain is enclosed within the skull and its expandability is restricted, the ability to compensate for pressure-volume changes (auto-regulation) is also limited (i.e. compliance is low). Under normal conditions, auto-regulatory processes are responsible for keeping the intracranial volume constant. As brain compliance starts to decrease, the compensatory capacity is exhausted so that further volume changes are no longer accommodated; this causes ICP to increase. Space-occupying lesions are the main causes for the changes in intracranial volume. Hydrocephalus, intracranial haemorrhage, haematoma, and brain edema are examples of such lesions [19].

Under normal compensatory adaptations, the ICP stays within a narrow pressure range for each assumed body posture [20, 21]. This is the simplest way of looking at ICP, as just a number that should remain within certain boundary values. Going beyond that, the ICP signal can be analyzed from a different perspective by studying the presence of macro-patterns. The diversity of B waves is the most commonly encountered macro-wave in clinical practice.

This study demonstrates the lack of agreement with regard to the terminology and characteristics used to define B waves. Different names are used to refer to the same phenomena, in order to either describe characteristics and morphological variations of the wave or the etiology behind their occurrence. This makes mathematical modeling of B waves more difficult, which consequently complicates the selection or development of an analysis tool that could be used to automatically interpret them. Automating B wave identification may be a way to detect and better understand ICP deviations from a normal physiological state at an earlier stage. But with the focus of current analysis tools on identifying previously defined B waves, they share a limitation of throwing away data related to other potentially relevant waveform deviations. Thus, underlying patterns of ICP that may contain important information on the interplay of physiological systems affecting the brain are potentially neglected. Opening up the analysis of ICP signals without being limited to previously defined patterns and conventions could enable fruitful new investigative and diagnostic techniques.

### Sub-classification

B waves were first defined from ICP monitoring sessions recorded in severely ill patients. Sub-classifications, which have mainly been qualitative, are the only attempts at modernizing the description of B waves to fit the clinical situations we see today [6–9].

The existence of multiple attempts at B wave subgrouping suggests that the overarching B wave category is not satisfactory for classification purposes today. A future avenue of research may instead be to direct attention away from classical B wave detection and instead focus on the identification of new parameters to automate the analysis of repeatable patterns in the ICP signal. Pattern recognition algorithms will be the fundamental approach used for this purpose.

### Analysis tools

ICP signals arise from the interaction of multiple physiological factors (e.g. heart pump, respiration, ...) that vary over time. Thus, it may be seen as a time series signal [22]. Traditionally, ICP signals have been inspected visually for B wave identification. In addition to being a time consuming technique, it is also subject to investigator bias due to interpretation subjectivity and dependence on clinical experience. Since the introduction of computerized algorithms, spectral analysis has led the way in B wave detection. A general agreement on a certain frequency range that this wave occupies may explain why spectral analysis is the most reported methodology. However, there is low frequency activity within the B-wave range that is unrelated to vasomotor activity (i.e. respiratory changes associated with sleep), thereby introducing a severe limitation in the use of spectral analysis. We might get unwanted contributions from these signals in the B-wave frequency range when breaking down the signal into frequency components. Eklund et al. developed an algorithm that strives to overcome this problem by also taking into account the wave amplitude [23].

Defining B waves in terms of amplitude is, however, very ambiguous. In particular, the term amplitude can be approached as the trough to peak pressure difference in the signal. If the wave has a sinusoidal appearance there is no problem in the identification of both its maximum and minimum values, but their identification becomes a challenge when the waveform is irregular. At the same time, the term amplitude can also refer to the distance from the peak of the wave to the baseline.

MOCAIP extracts morphological parameters from the pulse wave that are then used to characterize B waves instead of defining them based on their amplitude and frequency [24]. With the advantage of no longer depending on the classical B wave definition, this algorithm presents other drawbacks that prevents it from proper implementation in clinical practice. It rejects ICP pulses if a corresponding matching template is not included within the reference library proposed. This library is limited to intraparenchymal ICP signals from patients with hydrocephalus and does not comprise any ICP pulses from other pathologies, so that ICP pulses could be falsely rejected. Another limitation is the requirement of a simultaneous acquisition of ECG signal to help in the identification of the ICP pulse wave. Also, identifying B waves using MOCAIP assumes that the pulse waves are affected during the B waves, which is not definitively settled. Another approach proposed by Elixmann also isolates the pulse waves and classifies them based on predefined templates [25].

## Conclusion

To exploit the potential role of macro-patterns in ICP dynamics and to automate their identification for diagnostic or therapeutic purposes, two approaches for future work may be considered.

There could be efforts to arrive at strict agreement on morphological characteristics of classical macro-patterns, which requires consensus-based definitions to enable the derivation of relevant metrics to characterize them.

Alternatively, a new approach could be attempted without relying on classical macro-patterns. Instead it could be based on recognition of new patterns that more adequately describe variations seen in daily clinical practice today. This de novo pattern recognition approach requires relating macro-patterns to clinical information to ensure that they are biologically relevant.

## Data Availability

Data sharing is not applicable to this article as no datasets were generated or analysed during the current study.

## Abbreviations

ICP: intracranial pressure
CSF: cerebrospinal fluid
ICU: intensive care unit
MOCAIP: morphological clustering and analysis of ICP pulse
